# The Specificity and Patterns of Staining in Human Cells and Tissues of p16INK4a Antibodies Demonstrate Variant Antigen Binding

**DOI:** 10.1371/journal.pone.0053313

**Published:** 2013-01-08

**Authors:** Magdalena Sawicka, Jeffrey Pawlikowski, Stephen Wilson, Dudley Ferdinando, Hong Wu, Peter David Adams, David Andrew Gunn, William Parish

**Affiliations:** 1 Unilever Discover, Colworth Science Park, Sharnbrook, Bedford, United Kingdom; 2 Institute of Cancer Sciences, CR-UK Beatson Labs, University of Glasgow, Glasgow United Kingdom; 3 Fox Chase Cancer Center, Philadelphia, Pennsylvania, United States of America; King’s College London, United Kingdom

## Abstract

The validity of the identification and classification of human cancer using antibodies to detect biomarker proteins depends upon antibody specificity. Antibodies that bind to the tumour-suppressor protein p16INK4a are widely used for cancer diagnosis and research. In this study we examined the specificity of four commercially available anti-p16INK4a antibodies in four immunological applications. The antibodies H-156 and JC8 detected the same 16 kDa protein in western blot and immunoprecipitation tests, whereas the antibody F-12 did not detect any protein in western blot analysis or capture a protein that could be recognised by the H-156 antibody. In immunocytochemistry tests, the antibodies JC8 and H-156 detected a predominately cytoplasmic localised antigen, whose signal was depleted in p16INK4a siRNA experiments. F-12, in contrast, detected a predominately nuclear located antigen and there was no noticeable reduction in this signal after siRNA knockdown. Furthermore in immunohistochemistry tests, F-12 generated a different pattern of staining compared to the JC8 and E6H4 antibodies. These results demonstrate that three out of four commercially available p16INK4a antibodies are specific to, and indicate a mainly cytoplasmic localisation for, the p16INK4a protein. The F-12 antibody, which has been widely used in previous studies, gave different results to the other antibodies and did not demonstrate specificity to human p16INK4a. This work emphasizes the importance of the validation of commercial antibodies, aside to the previously reported use, for the full verification of immunoreaction specificity.

## Introduction

For the accurate diagnosis of human cancers, protein expression has been the focus of much attention due to the accuracy, sensitivity and ease with which antibodies can be used to detect the presence of proteins in tissue samples. P16INK4a, classified as an important tumour-suppressor protein, is a potent inhibitor of cell proliferation that mediates G1 cell cycle arrest through the regulation of Retinoblastoma (Rb) family of proteins [1], [2]. Inactivation of p16INK4a expression via point mutation, small deletion or promoter methylation has been reported in numerous types of human malignancies, resulting in it being extensively studied as a biomarker for cancer diagnosis and prognosis. Although some cancers are associated with a down-regulation of p16INK4a [3] others, particularly those associated with human papillomavirus infection, are associated with an increased expression. Currently, there is evidence that immunohistochemical tests for p16INK4a expression can be a valuable supplementary marker for cervical cancer diagnosis as p16INK4a protein is over-expressed in most cases of cervical dysplasia and invasive squamous cell carcinoma [4]–[7]. P16INK4A immunohistochemistry analysis of biopsy specimens has also been proposed as a prognostic test in cases of non-small cell lung cancer [8], differentiated thyroid cancer [9] and melanoma [10]. However, p16INK4a as a biomarker in cancer diagnostics has been most useful in conjunction with other biomarkers, particularly in supporting histological test for cervical cancer [6].

For an antibody to be used in a diagnostic kit, full validation of the antibody is essential to ensure its specificity and sensitivity to the target protein in the appropriate assay. For example, the specificity and sensitivity of the p16INK4a antibody E6H4 has been validated in human samples [4] and is now used in diagnostic kits for cervical cancer. However, most antibodies that are commercially available for research purposes are less well characterised.

We report the examination of four p16INK4a antibodies recommended for detection of human p16INK4a antigen that are either used extensively in western blot analyses (H-156), human tissue staining (JC8), bind to a specific nuclear isoform of p16INK4a (F-12) or considered a gold standard in cervical diagnostics (E6H4). The antibodies were examined by western blot analysis, immunoprecipitation (IP), immunohistochemistry (IHC) and immunocytochemistry (ICC) tests. Furthermore we examined the effect of p16INK4a siRNA knockdown on immunocytochemistry staining.

## Materials and Methods

### Antibodies

Anti-p16INK4a antibodies tested were mouse monoclonal F-12 (sc-1661, Santa Cruz Biotechnology) raised against full length p16INK4a of mouse origin, mouse monoclonal JC8 (sc-56330, Santa Cruz Biotechnology) against human full length p16, rabbit polyclonal H-156 (sc-759, Santa Cruz Biotechnology) against human full length p16INK4a and mouse monoclonal antibody clone E6H4 (CINtec Histology Kit, MTM Laboratories) raised against human p16INK4a protein.

### Western Blotting

Human embryonic kidney (Hek293) and cervix HeLa cell lysates were used as positive controls and whole cell PC-3 extract was used as a negative control as these cells do not express the p16INK4a protein [11]. HeLa and Hek293 cells (both cell lines purchased from American Type Culture Collection) were lysed in 1% Triton X-100 in PBS plus protease inhibitors cocktail (Sigma) and protein content was determined using BCA Protein Assay Kit (Thermo Scientific). PC3 whole cell lysate was purchased from Santa Cruz Biotechnology. Proteins were separated on the NuPage 4–12% bis-tris acrylamide gels (Invitrogen) in MES Buffer (Invitrogen) and transferred onto PVDF membrane (Invitrogen). After blocking with 2% skimmed milk powder in 0.1% Tween20/PBS (PBST), membranes were probed with primary antibodies in blocking buffer for 2 hours. Anti-p16INK4a antibodies H-156, JC8 and F-12 were used at the following concentrations: 0.4 µg/ml, 1 µg/ml, 1 µg/ml respectively. Blots were washed in PBST and incubated for 1 hour with the appropriate anti-mouse or anti-rabbit horseradish peroxidase-conjugated secondary antibodies (Jackson ImmunoResearch) diluted 1∶4000 in blocking buffer. Detection was via Super Signal West Pico Chemiluminescent Substrate (Thermo Scientific). Immunoreactive bands were visualized using ChemiDoc XRS imaging system and Quantity One analysis software (Bio-Rad).

### Immunoprecipitation

HeLa and Hek293 cell lysates containing 500 µg protein were incubated for 1 hour with 2 µg of the F12 or JC8 antibodies in a final volume of 1ml PBST (plus protease inhibitors cocktail). As a negative control mouse monoclonal anti-FLAG antibody was used in the same manner. Subsequently antibody-lysate mixtures were added to Sheep anti-Mouse IgG Dynabeads (Invitrogen) and incubated for 1 hour. Supernatants were collected and beads washed 5 times with PBST. Protein-antibody complexes were eluted from the beads by adding 70 µl of 0.1M glycine/HCl pH 2.6. 10 µl of each IP sample was mixed with LDS buffer and run on gels as described for Western blot analysis. Polyclonal rabbit anti-p16INK4a H-156 antibody was used to detect immunoprecipitated p16.

### Immunocytochemistry

HeLa cells, purchased from American Type Culture Collection, were cultured in Dulbecco’s modified Eagle medium (Invitrogen) supplemented with 10% fetal bovine serum and 1 mM sodium pyruvate. For ICC experiments cells were seeded at 1×10^5^ per well on glass coverslips in 6-well plates and allowed to attach for 24 hours. Following fixation in 2% paraformaldehyde for 20 minutes, PBS wash, and permeabilization with 0.5% saponin in PBS, cells were blocked for 1 hour with 0.2% bovine serum albumin in PBS and subsequently incubated with primary antibodies for two hours. Anti-p16INK4a antibodies H-156, F-12 and JC8 were used at the following concentrations: 1 µg/ml, 1 µg/ml and 2 µg/ml respectively and E6H4 as received. Cells were washed four times with 0.2% BSA in PBS and incubated for two hours with secondary antibodies. Secondary antibodies were Alexa Fluor 488 donkey anti-rabbit, Alexa Fluor 488 donkey anti-mouse and Alexa Fluor 633 donkey anti-sheep (Invitrogen). Cover-slips were mounted using ProLong Gold Antifade Reagent. For confocal microscopy a Leica TCSSP1 confocal scanning laser microscope with Leica LCS software was used. Images were acquired using a 63×1.4 n.a oil immersion objective. 488 nm and 633 nm laser excitation wavelengths were utilised.

### Immunohistochemistry

Formalin fixed paraffin embedded breast skin, diagnosis unknown, was sourced from Source BioScience (formerly Medical Solutions) UK, and three cervical samples (one adenocarcinoma, one squamous cell carcinoma [SCC] and one keratinising SCC) were sourced from TissueSolutions UK. Sections of benign nevi were obtained from Fox Chase Cancer Center (Philadephia, USA); nevi were designated benign after histological examination by a pathologist. In order to detect p16INK4a in the tissues, CINtec clinical test reagents were used according to the manufacturers’ instructions (CINtec Histology Kit, MTM Laboratories). Serial sections were treated for 1 hour with one of the following, control no antibody solution (CINtec kit), anti-p16INK4a antibodies: E6H4 as received (CINtec kit), F-12 at 0.4 µg/ml and JC8 at 0.4 µg/ml diluted in 2% bovine serum albumin in TBS. Sections were counterstained with Mayer’s hematoxylin, washed and mounted in glycergel (DAKO).

### siRNA Mediated p16INK4a Knockdown

HeLa cells, purchased from American Type Culture Collection, were transfected using 2 µg/ml lipofectamine 2000 (Invitrogen) and 40 nM of siRNA probe 403 (Qiagen code SI02664403) or 817 (Qiagen code SI00299817) in Opti-MEM medium (Invitrogen) for 6 hours. Cells were returned to growth medium for 72 hours before immunofluorescence analysis. Lipofectamine alone and non-targeting siRNA AllStars (Qiagen) were used as negative controls.

## Results

**Figure 1:**
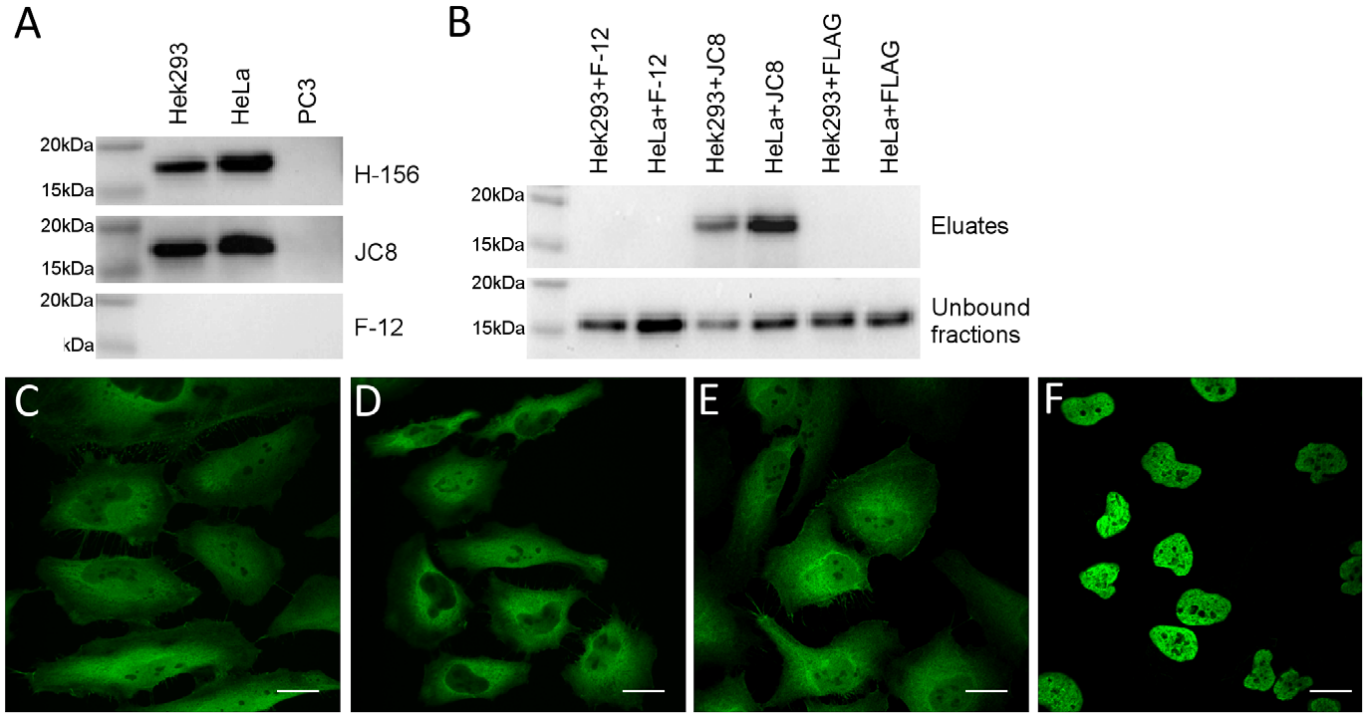
P16INK4A antibody validation. P16INK4A subcellular localization by immunofluorescence in HeLa cells using anti-p16INK4a antibodies F-12 (**A**), H-156 (**B**), JC8 (**C**), and E6H4 (**D**); magnification bar  = 20 µm. **E**. Comparison of anti-p16INK4a antibodies in Western Blot analysis using p16INK4a positive Hek293 and HeLa whole cell lysates and p16INK4a negative PC3 cell extract (each loaded 20 µg whole protein per well). **F**. Immunoprecipitation of p16INK4a using F-12 and JC8 mouse monoclonal antibodies, the FLAG mouse monoclonal antibody was used alongside as a negative control.

To characterise anti-p16INK4a antibody specificity, we first carried out western blot analysis. Both the rabbit polyclonal H-156 and mouse monoclonal JC8 antibodies detected a single band of appropriate molecular mass for p16INK4a (approximately 16 kDa) in HeLa and Hek293 cell lysates. No such staining was detected in lysate from PC-3 cells, in which expression of p16INK4a is silenced by promoter methylation [11]. In contrast, the F-12 antibody did not detect any band in the western blot application (Figure 1A). The F-12 and JC8 were then tested for their ability to immunoprecipitate native p16INK4a protein from human HeLa and Hek293 cell lysates. As shown in Figure 1B, the JC8 antibody was able to specifically immunoprecipitate antigens from HeLa and from Hek293 cell lysates that were recognised by the H-156 antibody and were approximately 16 kDa in size. F-12, in contrast, did not bind to any antigen recognised by the H-156 polyclonal antibody in this test.

**Figure 2:**
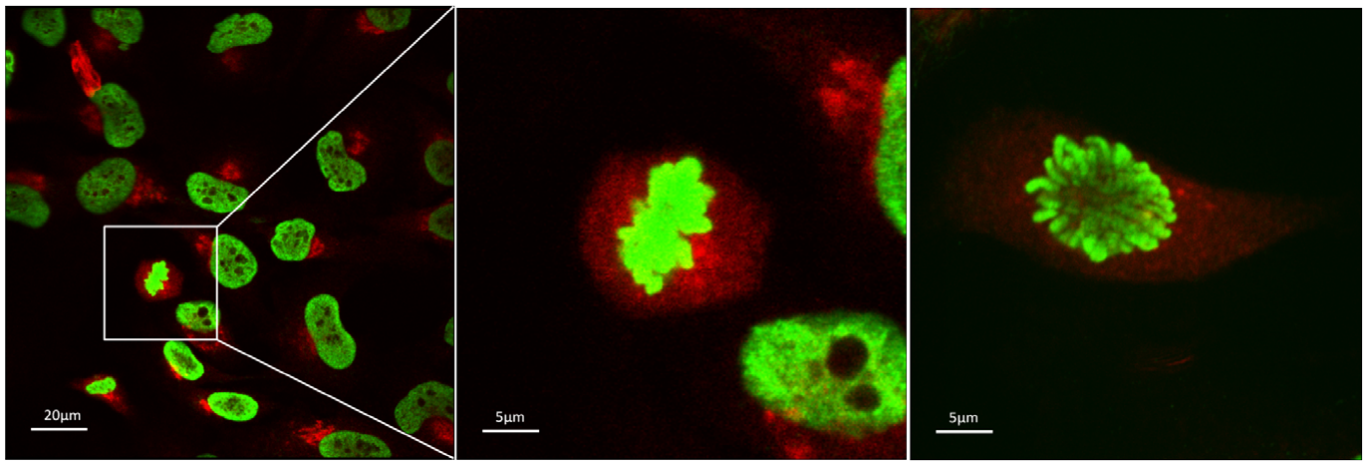
The F12 antibody localisation to the chromosomes. HeLa cells were fixed and double-labelled by immunofluorescence with anti-p16INK4a F12 antibody (green) and anti-TGN antibody (red). The F12 antibody localised to the chromosomes during the metaphase (**A**) and early anaphase (**B**) of cell division.

**Figure 3:**
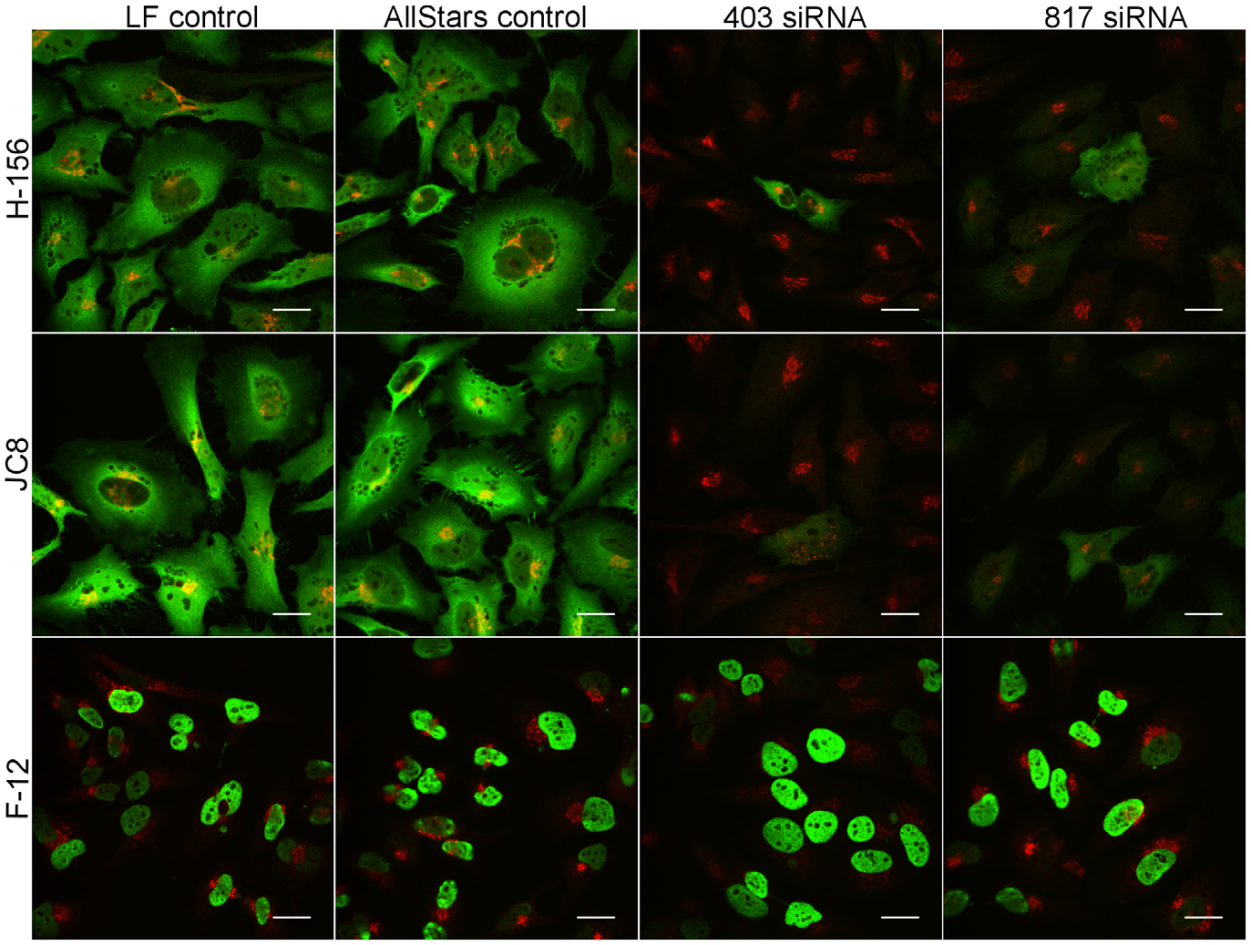
P16INK4A siRNA knockdown. HeLa cells were transfected with targeting siRNA duplexes 403 and 817, and non-targeting siRNA control duplex AllStars or Lipofectamine 2000 alone. 72 hours post-knockdown cells were fixed and double-labelled with the anti-p16INK4a antibodies (green) H-156, JC8, F-12 and with the TGN46 antibody (red) along with the appropriate secondary AlexaFluor antibodies. Images were separately recorded in the red and green by immunofluorecsence confocal microscopy and merged. Magnification bar  = 20 µm.

The anti-p16INK4a antibodies JC8, E6H4 and H-156 showed very similar patterns of staining in ICC tests (Figure 1C–1E). Although the HeLa cell staining was predominately cytoplasmic, there was some evidence of weak nuclear staining. In contrast, the F-12 antibody staining showed strong immunoreactivity in the nucleus with little evidence of cytoplasmic staining (Figure 1F). The variant antibody binding between F-12 and JC8 was also observed using two different fixation techniques (Figure S1). Additionally, F12 localisation to the chromosomes during the metaphase and anaphase of cell division was observed (Figure 2). We then used siRNA mediated gene knockdown followed by immunofluorescence with the H-156, JC8 and F-12 antibodies; a counter stain to the trans-Golgi network (antibody TGN46) was also used to aid cellular localisation. As shown in Figure 3, the intensity of staining was markedly reduced in siRNA treated cells when assessed by the H-156 and JC8 antibody stains. In contrast, the F-12 antibody staining demonstrated only moderate depletion of nuclear immunoreactivity, and this was also observed in the AllStars siRNA and Lipofectamine controls.

**Figure 4:**
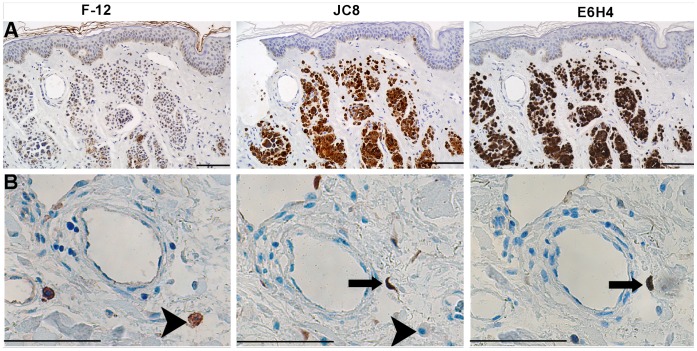
IHC analysis of nevi and breast skin samples. Serial sections of a nevi skin sample (A) and a breast skin sample (B) stained immunohistochemically with the F-12 (left-hand column), JC8 (middle column) and E6H4 (right hand column) antibodies to enable field to field comparison. The E6H4 and JC8 antibodies, but not the F-12 antibody, gave qualitatively very similar patterns of staining. The E6H4 and JC8 detected numerous nevus melanocytes (brown staining) in the dermis, whereas only a fraction of these were weakly detected by F-12 (A). Whilst the JC8 and E6H4 antibodies detected fibroblasts (arrows) in a breast sample (B) the F-12 antibody gave cytoplasmic staining in round mononuclear cells which were undetected with the JC8 antibody (arrowheads). The breast and nevi experiments were carried out in independent laboratories using different antibody lots/batches. Magnification bar  = 100 µm.

**Figure 5:**
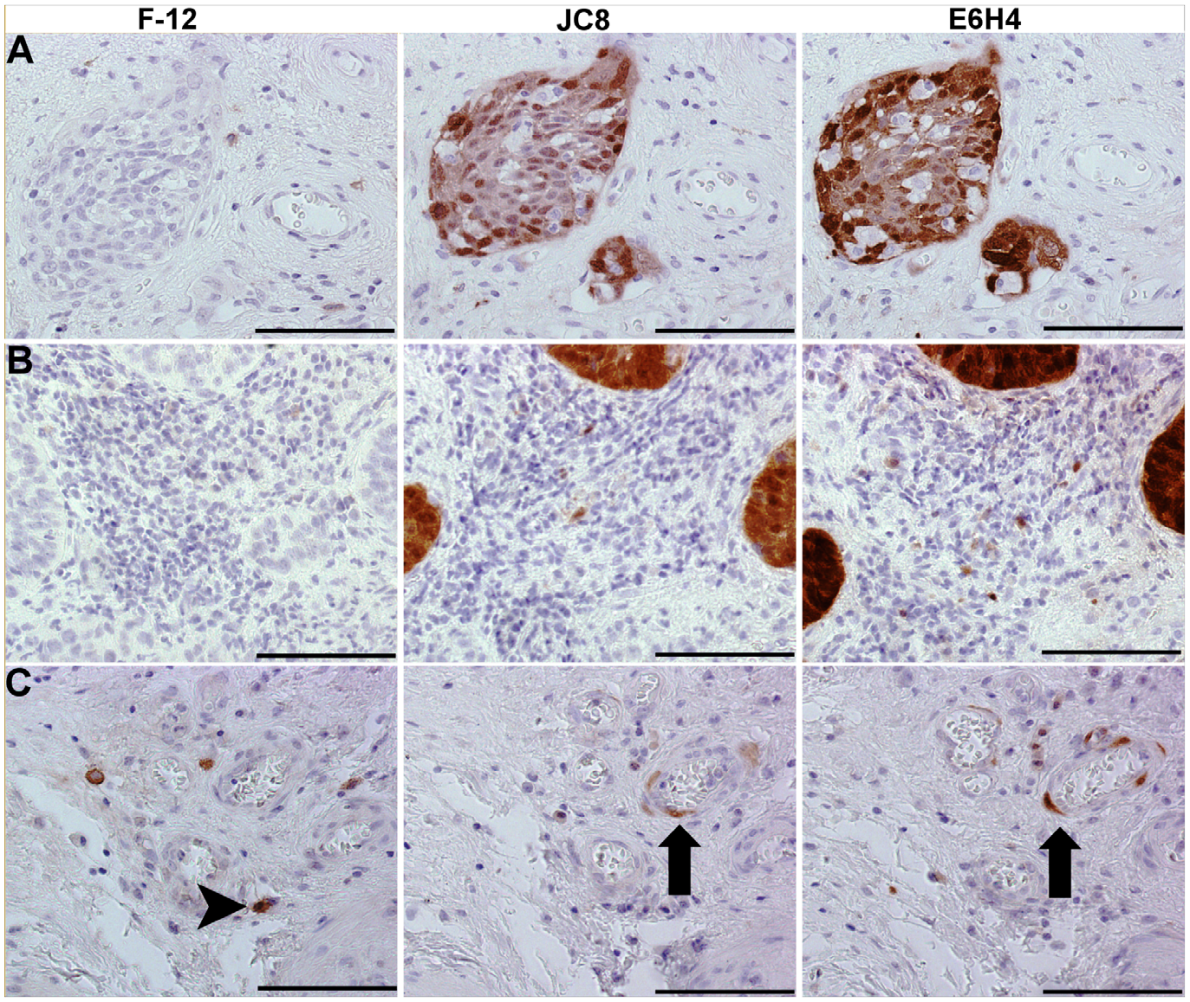
IHC analysis of cervical samples. Serial sections of three cervical SCC samples stained immunohistochemically with the F-12 (left-hand column), JC8 (middle column) and E6H4 (right hand column) antibodies to enable field to field comparisons. F-12 failed to stain neoplastic structures clearly detected by JC8 and E6H4 in SCC (A) and keratinising SCC (B) samples, and gave a different pattern of cellular staining in tissue peripheral to the neoplastic mass in an adenocarcinoma cervical sample (compare arrows and arrowheads in C). Magnification bar  = 100 µm. For a comparative negative control sections, see Figure S2.

To determine whether the p16INK4a antibodies give similar staining patterns in human tissue, IHC analysis of the F-12 and JC8 antibodies was performed on serial tissue sections alongside the antibody E6H4 (Figure 4) which is commonly used for the identification of p16INK4a in cervical cancer tissue in IHC tests [12]. Within human skin, melanocytes of benign nevi (moles) are senescent due to their expression of a mutated and activated N-RAS or BRAF oncogene [13]. Consequently, many of these cells express p16INK4a [13]. In addition, the epidermis of skin from elderly individuals has been demonstrated to harbour isolated p16INK4a-positive cells [14]. The JC8 and E6H4 antibodies generated virtually identical staining patterns in nevus samples, whereas F-12 gave a different pattern of staining (Figure 4A). JC8 and E6H4 stained the senescent dermal nevus melanocytes, as reported previously [13]. Nevus melanocytes were confirmed by melanA staining (data not shown) and histologically by a qualified dermatopathologist (author HW). Both antibodies stained weakly in the epidermis, with some isolated positive cells, as reported previously [13], [14]. In contrast, F-12 stained relatively weakly in the nevus melanocytes. Moreover, the ratio of diffuse epidermal to nevus stain was higher for F12 compared to JC8 and E6H4, and F12 also largely lacked the isolated p16INK4a-positive cells. We also found that the F-12 gave a different pattern of staining to JC8 and E6H4 in a breast skin sample (Figure 4B) supporting the notion that F-12 was binding to a different antigen to the other two antibodies in the IHC tests. Finally, to determine the relevance of using these antibodies for cervical diagnostics, we then stained serial sections from three different cervical samples. Whilst JC-8 and E6H4 detected cervical neoplastic cells (Figure 5A and B), the F-12 antibody only stained small numbers of cells peripheral to the tumours (Figure 5C); for a negative control section, see Figure S2.

## Discussion

The p16INK4a protein is 16 kDa in size and consists of 156 amino-acids [2]. In western blot tests presented here, the p16INK4a antibodies JC8 and H156 detected a protein of the expected size, whereas F-12 failed to detect any protein. In addition, the JC8 antibody captured a 16 kDa protein that H156 was able to detect in western blot analysis, whereas with the same protocol F-12 did not capture any protein that was recognised by H156. The F-12 antibody was raised against the full length p16INK4a protein from mouse. The high degree of amino acid homology (85%) between p16INK4a in human and mouse predicts that this antibody might be suitable for work in human tissue and three previous studies have indicated that F-12 is capable of binding to a 16 kDa human protein in western blot analysis [15], [16], [17]. However, our data indicates that the F-12 antibody, compared to the H156 and JC8 antibodies, has a greatly reduced sensitivity for detection of a 16 kDa human protein in western blot analysis. This is supported by comparable western blot images for the JC-8 and F-12 antibodies presented in one of the three previous reports [17] which illustrate a highly reduced F-12 antibody sensitivity for detecting a 16 kDa protein.

A predominant cytoplasmic signal was found for the antibodies H156, E6H4 and JC8 in ICC tests. In addition, the cytoplasmic signal detected by H156 and JC8 was lost in siRNA mediated knockdown of p16INK4a. This demonstrates that the antibodies H-156 and JC8 have good specificity to human p16INK4a. In contrast, the F-12 antibody had a predominately nuclear stain with little, if any, staining in the cytoplasm of the HeLa cells. Two forms of the human p16INK4a protein have been detected in 2D electrophoresis experiments, with one form mainly located in the nucleus and the other in the cytoplasm [18]. Whilst some reports have located p16INK4a predominately to the cytoplasm of a range of cell lines [4], [19], two reports located p16INK4a predominantly to the nucleus of the WI-38 fibroblast cell line [3], [20]. Hence, it is plausible that F-12 is binding to a nuclear form of p16INK4a and the other antibodies to the cytoplasmic form. This notion has also been recently proposed by Haller et al. [17] who demonstrated that the F-12 nuclear stain had prognostic value (albeit less than the JC8 cytoplasmic stain) in gastrointestinal stromal tumours (GISTs). In addition, they found that loss of the 9P chromosomal region (which includes the p16INK4a locus) in GISTs was associated with a decreased F-12 immunoreactivity, suggestive that the F-12 antigen originates from a gene in this region [21]. If true, it is intriguing that F-12 also localised to the chromosomes during the metaphase and anaphase of cell division as such a localisation for the p16INK4a protein has not been previously reported. Most concerning, in ICC assays, staining by F-12 was not abolished by siRNA knock down of p16INK4a, supporting the notion that, at least under these conditions, the F-12 staining is non-specific.

In IHC tests, the F-12 antibody gave variant binding patterns compared to JC8 and E6H4 across different F-12 antibody lots and in two different laboratories (Figure 4); in addition, differences in tissue patterns between JC8 and F-12 has also been reported elsewhere [17]. Together, these data indicate it was highly unlikely that the differences in staining between antibodies were due to purely methodological reasons. Although F-12 was predominately located in the nucleus in ICC tests, this was not the case in IHC tests indicating that F-12 was not binding to a protein isoform that is exclusive to the nucleus in human tissues.

Taken together with the western blot data, these data indicate that the F-12 antibody might not be binding to the human p16INK4a protein. This raises doubt on the conclusions of a number of studies that have used F-12 (all from Santa Cruz) to detect the p16INK4a protein in human cancer tissues (24 manuscripts identified, Supporting Information S1). In most cases the F-12 antibody was used to correlate the level of p16INK4a expression in cancer with prognosis. Some authors have reported a correlation of altered F-12 staining with worse patient outcome in lung cancer [22], although others found no such correlation [23]. Moreover, it has been reported that p16INK4a inactivation, assessed with F-12 antibody, was involved in development and progression of other malignancies such as nasopharyngeal carcinoma [24], thick melanomas [25], and adrenocortical tumours [26]. While we can not eliminate that this antibody did bind to an isoform of the human p16INK4a protein in our tests or in the previously reported studies, further investigations are required before the reported F-12 data can be attributed with certainty to the human p16INK4a protein. In conclusion, these data pin-point the localisation of p16INK4a in human cells and tissue, validates the specificity of three p16INK4a antibodies and highlights the importance of specificity validation for commercially available antibodies.

## Supporting Information

Figure S1 ICC analysis with different fixation techniques. HeLa cells grown on glass coverslips were fixed either with 2% paraformaldehyde (PFA) for 20 minutes or with ice-cold methanol-acetone (M/A) mixture (1∶1) for 5 minutes and immunofluorescently labelled with ant-p16^INK4A^ antibodies F12 and JC8 as described in Materials and Methods. Slight differences in antibody staining for both F12 and JC8 antibodies are seen between different fixatives, immunofluorescent signal is more intense in cells fixed with PFA. It is also apparent in phase-contrast images that alcohol fixative did not preserve cell morphology as well as paraformaldehyde. Therefore the difference in intensity of immunolabelling can be explained as effect of morphological changes rather than any alterations in antibody staining pattern under different fixation methods. Moreover, with both fixatives, the F-12 antibody shows only nuclear immunoreactivity whereas the JC8 antibody stains nuclear and cytoplasmic antigen.
(TIF)

Figure S2 P16INK4a antibody staining of a cervical sample including negative control. No primary antibody (left-hand image), F-12 (second from left), JC8 (second from right) and E6H4 (right-hand image) staining of serial sections of a cervical sample; brown cellular staining is evident in all images but the negative control.
(TIF)

Supporting Information S1 References for the use of F12 antibody.
(DOC)
