# Supplementary figures and images for: The Specificity and Patterns of Staining in Human Cells and Tissues of p16INK4a Antibodies Demonstrate Variant Antigen Binding
Source: PLoS One. 2013 Jan 8;8(1):e53313. doi: 10.1371/journal.pone.0053313 (PMC3540092; doi:10.1371/journal.pone.0053313)

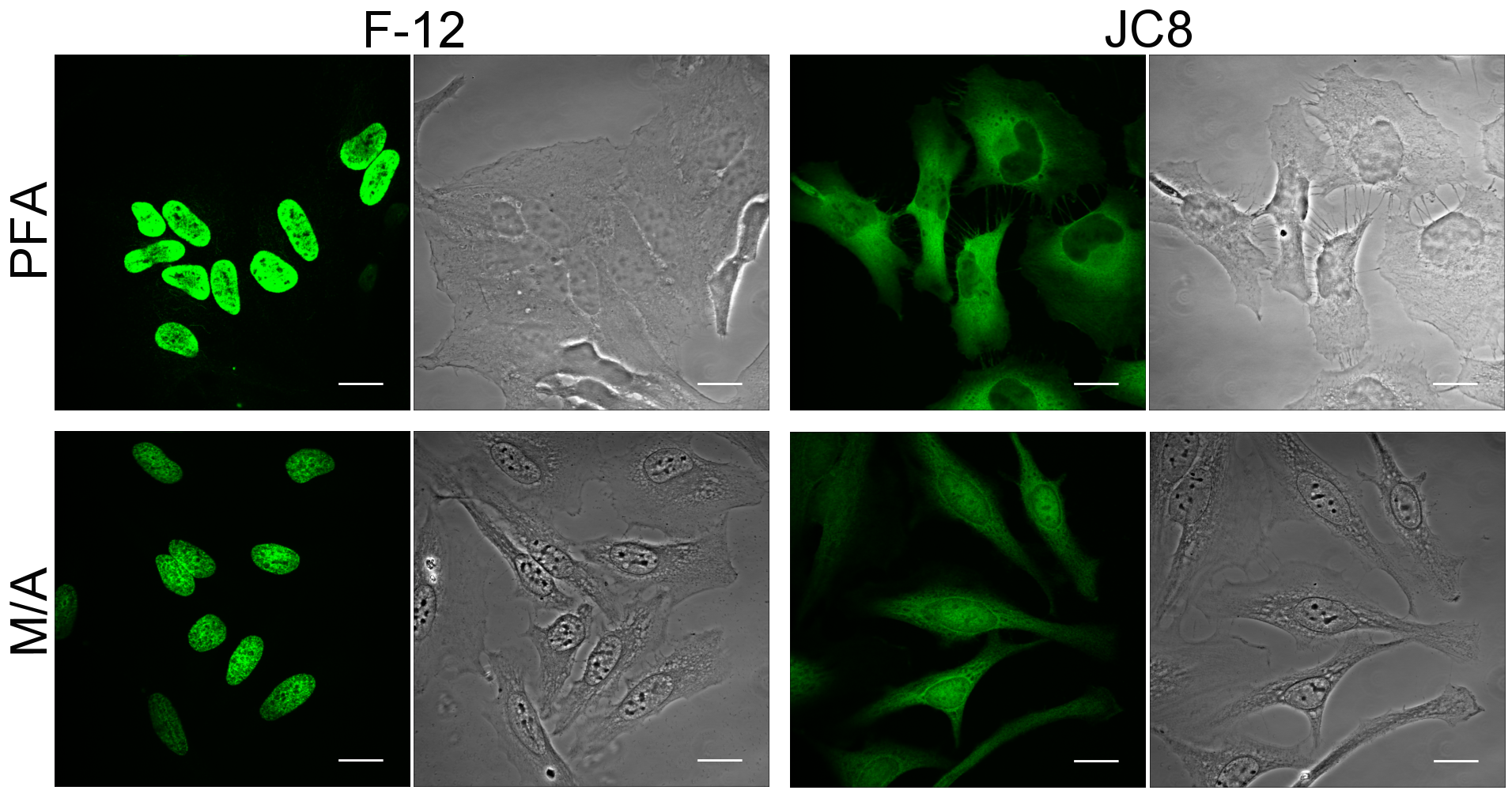

Supplement: Figure S1 — ICC analysis with different fixation techniques. HeLa cells grown on glass coverslips were fixed either with 2% paraformaldehyde (PFA) for 20 minutes or with ice-cold methanol-acetone (M/A) mixture (1∶1) for 5 minutes and immunofluorescently labelled with ant-p16INK4A antibodies F12 and JC8 as described in Materials and Methods. Slight differences in antibody staining for both F12 and JC8 antibodies are seen between different fixatives, immunofluorescent signal is more intense in cells fixed with PFA. It is also apparent in phase-contrast images that alcohol fixative did not preserve cell morphology as well as paraformaldehyde. Therefore the difference in intensity of immunolabelling can be explained as effect of morphological changes rather than any alterations in antibody staining pattern under different fixation methods. Moreover, with both fixatives, the F-12 antibody shows only nuclear immunoreactivity whereas the JC8 antibody stains nuclear and cytoplasmic antigen. (TIF) [file pone.0053313.s001.tif]

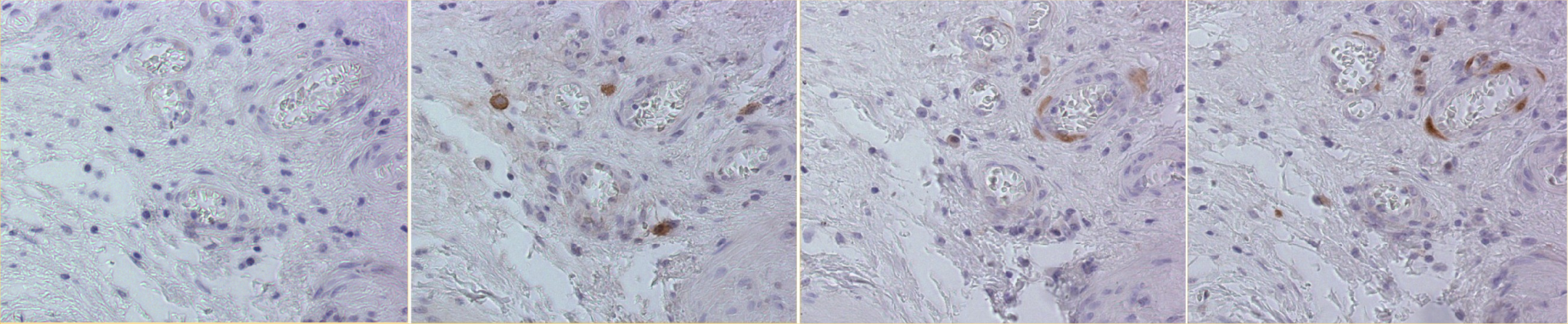

Supplement: Figure S2 — P16INK4a antibody staining of a cervical sample including negative control. No primary antibody (left-hand image), F-12 (second from left), JC8 (second from right) and E6H4 (right-hand image) staining of serial sections of a cervical sample; brown cellular staining is evident in all images but the negative control. (TIF) [file pone.0053313.s002.tif]
