# Supplementary material for: The Specificity and Patterns of Staining in Human Cells and Tissues of p16INK4a Antibodies Demonstrate Variant Antigen Binding
Source: PLoS One. 2013 Jan 8;8(1):e53313. doi: 10.1371/journal.pone.0053313 (PMC3540092; doi:10.1371/journal.pone.0053313)
Supplement: Supporting Information S1 — References for the use of F12 antibody. (DOC) [file pone.0053313.s003.doc]

## References for the use of the F-12 antibody.

1. Groeger AM, Caputi M, Esposito V, De Luca A, Bagella L, et al. (1999) Independent prognostic role of p16 expression in lung cancer. J. Thorac Cardiovasc Surg. 118(3): 529-35.
2. Seike M, Gemma A, Hosoya Y, Hemmi S, Taniguchi Y, et al. (2000) Increase in the frequency of p16INK4 gene inactivation by hypermethylation in lung cancer during the process of metastasis and its relation to the status of p53. Clin Cancer Res. 6(11): 4307-13.
3. Valbuena A, Suárez-Gauthier A, López-Rios F, López-Encuentra A, Blanco S, et al. (2007) Alteration of the VRK1-p53 autoregulatory loop in human lung carcinomas. Lung Cancer. 58(3): 303-9.
4. Rodriguez-Pinilla M, Rodriguez-Peralto JL, Hitt R, Sanchez JJ, Ballestin C, et al. (2004) Cyclin A as a predictive factor for chemotherapy response in advanced head and neck cancer. Clin Cancer Res. 10(24): 8486-92.
5. Lin HS, Berry GJ, Sun Z, Fee WE Jr (2006) Cyclin D1 and p16 expression in recurrent nasopharyngeal carcinoma. World J Surg Oncol. 4: 62.
6. Noguera R, Machado I, Piqueras M, Lopez-Guerrero JA, Navarro S, et al. (2008) Tissue microarrays: applications in study of p16 and p53 alterations in Ewing's cell lines. Diagn Pathol. 3 (Suppl 1): S27.
7. Korkolopoulou P, Christodoulou P, Lazaris A, Thomas-Tsagli E, Kapralos P, et al. (2001) Prognostic implications of aberrations in p16/pRb pathway in urothelial bladder carcinomas: a multivariate analysis including p53 expression and proliferation markers. Eur Urol. 39(2): 167-77.
8. Chen JT, Chen YC, Chen CY, Wang YC (2001) Loss of p16 and/or pRb protein expression in NSCLC. An immunohistochemical and prognostic study. Lung Cancer. 31(2-3): 163-70.
9. Cheng YL, Lee SC, Harn HJ, Chen CJ, Chang YC, et al. (2003) Prognostic prediction of the immunohistochemical expression of p53 and p16 in resected non-small cell lung cancer. Eur J Cardiothorac Surg. 23(2): 221-8.
10. Pilon C, Pistorello M, Moscon A, Altavilla G, Pagotto U, et al. (1999) Inactivation of the p16 tumor suppressor gene in adrenocortical tumors. J Clin Endocrinol Metab. 84(8): 2776-9.
11. Brantley MA Jr, Harbour JW (2000) Inactivation of retinoblastoma protein in uveal melanoma by phosphorylation of sites in the COOH-terminal region. Cancer Res. 60(16): 4320-3.
12. Malanchi I, Accardi R, Diehl F, Smet A, Androphy E, et al. (2004) Human papillomavirus type 16 E6 promotes retinoblastoma protein phosphorylation and cell cycle progression. J Virol. 78(24): 13769-78.
13. Jang TJ, Kim DI, Shin YM, Chang HK, Yang CH (2001) p16(INK4a) Promoter hypermethylation of non-tumorous tissue adjacent to gastric cancer is correlated with glandular atrophy and chronic inflammation. Int J Cancer. 93(5): 629-34.
14. Taube ME, Liu XW, Fridman R, Kim HR (2006) TIMP-1 regulation of cell cycle in human breast epithelial cells via stabilization of p27(KIP1) protein. Oncogene. 25(21): 3041-8.
15. Wang JL, Zheng BY, Li XD, Angström T, Lindström MS, et al. (2004) Predictive significance of the alterations of p16INK4A, p14ARF, p53, and proliferating cell nuclear antigen expression in the progression of cervical cancer. Clin Cancer Res. 10(7): 2407-14.
16. De Sá BC, Fugimori ML, Ribeiro Kde C, Duprat Neto JP, Neves RI, et al. (2009) Proteins involved in pRb and p53 pathways are differentially expressed in thin and thick superficial spreading melanomas. Melanoma Res. 19(3): 135-41.
17. Marra DE, Simoncini T, Liao JK (2000) Inhibition of vascular smooth muscle cell proliferation by sodium salicylate mediated by upregulation of p21(Waf1) and p27(Kip1). Circulation. 102(17): 2124-30.
18. Brantley MA Jr, Harbour JW (2000) Inactivation of retinoblastoma protein in uveal melanoma by phosphorylation of sites in the COOH-terminal region. Cancer Res. 60(16): 4320-3.
19. Evangelou K, Bramis J, Peros I, Zacharatos P, Dasiou-Plakida D, et al. (2004) Electron microscopy evidence that cytoplasmic localization of the p16(INK4A) "nuclear" cyclin-dependent kinase inhibitor (CKI) in tumor cells is specific and not an artifact. A study in non-small cell lung carcinomas. Biotech Histochem. 79(1): 5-10.
20. Elenitoba-Johnson KS, Gascoyne RD, Lim MS, Chhanabai M, Jaffe ES, et al. (1998) Homozygous deletions at chromosome 9p21 involving p16 and p15 are associated with histologic progression in follicle center lymphoma. Blood. 91(12): 4677-85.
21. Gorgoulis VG, Koutroumbi EN, Kotsinas A, Zacharatos P, Markopoulos C, et al. (1998) Alterations of p16-pRb pathway and chromosome locus 9p21-22 in sporadic invasive breast carcinomas. Mol Med. 4(12): 807-22.
22. Nakamura M, Sakaki T, Hashimoto H, Nakase H, Ishida E, et al. (2001) Frequent alterations of the p14(ARF) and p16(INK4a) genes in primary central nervous system lymphomas. Cancer Res. 61(17): 6335-9.
23. Haller F, Lobke C, Ruschhaupt M, Cameron S, Schulten HJ, et al. (2008) Loss of 9p leads to p16INK4A down-regulation and enables RB/E2F1-dependent cell cycle promotion in gastrointestinal stromal tumours (GISTs). J Pathol. 215(3): 253-262.
24. Haller F, Agaimy A, Cameron S, Beyer M, Gunawan B, et al. (2010) Expression of p16INK4A in gastrointestinal stromal tumours (GISTs): two different forms exist that independently correlate with poor prognosis. Histopathology. 56(3): 305-318.
